# Adaptations in Maternofetal Calcium Transport in Relation to Placental Size and Fetal Sex in Mice

**DOI:** 10.3389/fphys.2017.01050

**Published:** 2017-12-12

**Authors:** Christina E. Hayward, Lewis J. Renshall, Colin P. Sibley, Susan L. Greenwood, Mark R. Dilworth

**Affiliations:** ^1^Division of Developmental Biology and Medicine, School of Medical Sciences, Faculty of Biology, Medicine and Health, The University of Manchester, Manchester, United Kingdom; ^2^Maternal and Fetal Health Research Centre, Manchester Academic Health Science Centre, St. Mary's Hospital, Central Manchester University Hospitals NHS Foundation Trust, Manchester, United Kingdom

**Keywords:** placenta, fetal growth restriction, calcium, IUGR, adaptation

## Abstract

Appropriate placental transport of calcium is essential for normal fetal skeletal mineralization. In fetal growth restriction (FGR), the failure of a fetus to achieve its growth potential, a number of placental nutrient transport systems show reduced activity but, in the case of calcium, placental transport is increased. In a genetic mouse model of FGR this increase, or adaptation, maintains appropriate fetal calcium content, relative to the size of the fetus, despite a small, dysfunctional placenta. It is unknown whether such an adaptation is also apparent in small, but normally functioning placentas. We tested the hypothesis that calcium transfer would be up-regulated in the lightest vs. heaviest placentas in the same C57Bl/6J wild-type (WT) mouse litter. Since lightest placentas are often from females, we also assessed whether fetal sex influenced placental calcium transfer. Placentas and fetuses were collected at embryonic day (E)16.5 and 18.5; the lightest and heaviest placentas, and female and male fetuses, were identified. Unidirectional maternofetal calcium clearance (^Ca^K_mf_) was assessed following ^45^Ca administration to the dam and subsequent radiolabel counts within the fetuses. Placental expression of calcium pathway components was measured by Western blot. Data (median) are lightest placenta expressed as percentage of the heaviest within a litter and analyzed by Wilcoxon signed-rank test. In WT mice having normally grown fetuses, ^Ca^K_mf_, per gram placenta near term, in the lightest placentas was increased (126%; *P* < 0.05) in association with reduced fetal calcium accretion earlier in gestation (92%; *P* < 0.05), that was subsequently normalized near term. Increased placental expression of calbindin-D_9K_, an important calcium binding protein, was observed in the lightest placentas near term (122%; *P* < 0.01). There was no difference in fetal calcium accretion between male and female littermates but a trend toward higher ^Ca^K_mf_ in females (*P* = 0.055). These data suggest a small, normal placenta adapts calcium transfer according to its size, as previously demonstrated in a mouse model of FGR. Fetal sex had limited influence on this adaptive increase. These adaptations are potentially driven by fetal nutrient demand, as evidenced by the normalization of fetal calcium content. Understanding the regulatory mechanisms involved may provide novel avenues for treating placental dysfunction.

## Introduction

Calcium is crucial for fetal bone mineralisation and maternofetal transport of calcium increases towards term to meet fetal requirements (Strid and Powell, 2000). Transcellular transport of calcium across the placenta has 3 major components: (i) movement from maternal blood, down an electrochemical gradient, into the cytosol of the syncytiotrophoblast (transporting epithelium of the placenta) through epithelial calcium channels (e.g., TRPV6, transient receptor potential vanniloid type 6), located on the maternal-facing microvillous membrane (MVM) (Bernucci et al., 2006; Haché et al., 2011; Yang et al., 2013) (ii) buffering by calcium binding proteins such as calbindin-D_9K_ in the trophoblast cytosol and delivery to the fetal-facing basal membrane (BM); and (iii) efflux of calcium across the BM against the electrochemical gradient mediated by the plasma membrane calcium ATPase (PMCA). Active placental calcium transport from mother to fetus in normal pregnancy maintains a higher calcium concentration in fetal compared to maternal plasma (Strid and Powell, 2000).

In human pregnancies complicated by fetal growth restriction (FGR), the activity of many placental nutrient transporters, including System A and System L amino acid transporters, is reduced, per gram placenta (Glazier et al., 1997; Jansson et al., 1998; Shibata et al., 2008). In contrast, the activity of PMCA on the BM is increased (Strid et al., 2003). This indicates that up-regulation of PMCA activity in FGR is a functional adaptation of the human placenta to try and ensure adequate fetal calcium acquisition despite placental pathology (Strid et al., 2003).

In support of this, our previous studies in a model of late onset FGR, the placental-specific insulin-like growth factor 2 knock out (P0) mouse (Constância et al., 2005), demonstrated that in placentas of growth restricted (P0), compared to normally grown (WT) fetuses, there is an increase in calcium transport near term as a response to reduced total fetal calcium and placental calbindin-D_9k_ protein expression earlier in gestation (Dilworth et al., 2010). Normalization of both fetal calcium content and calbindin-D_9k_ expression in P0 mouse fetuses suggests placental supply capacity can be matched to fetal nutrient demand and, despite being small and dysfunctional, P0 placentas are able to adapt their calcium transport to ensure appropriate calcium provision relative to the size of the fetus. What is not known is whether this adaptive increase in placental calcium transport in human and mouse FGR is FGR-specific, or whether such an adaptation also occurs in small placentas of normal pregnancy to maintain appropriate fetal growth.

In mice, the effect of placental size on maternofetal nutrient transfer can be assessed by comparing the lightest and heaviest placentas within a wild-type (WT) litter, as reported previously for System A and glucose (Coan et al., 2008). However, placentas of females in both mice and women, are, on average, lighter than those of males (Ishikawa et al., 2006; Misra et al., 2009; Almog et al., 2011; Wallace et al., 2013) and so one may expect a skew of female vs. males in terms of the lightest placenta group, with the opposite trend likely in the heaviest placentas. There is mounting evidence that in human pregnancies fetal sex has a major impact not only on fetal growth (*in utero* male fetuses grow bigger at an earlier gestation and have altered fetal biometric indices resulting in a larger birthweight than female fetuses; De Zegher et al., 1999; Melamed et al., 2013) but also on placental function and response to the intrauterine environment (reviewed by Clifton, 2010). It is therefore important to elucidate the effect of fetal sex, as well as placental size *per se*, on placental adaptations.

Here, we tested the hypothesis that to maintain appropriate fetal calcium acquisition and fetal growth, in WT mice, the lightest placentas in a litter of normally grown fetuses would demonstrate increased maternofetal calcium transport near term, per gram placenta, as compared to the heaviest placentas in the same litter and this effect would be independent of fetal sex.

## Materials and methods

### Ethical approval

Experiments were performed in accordance with UK Animals (Scientific Procedures) Act of 1986, under the authority of a UK Home Office project license (PPLs 40/3385 and 70/8504), and were authorized by the Animal Welfare and Ethical Review Board of the University of Manchester. The methods stated in this section adhere to the ARRIVE guidelines (Kilkenny et al., 2010) and comply with the animal ethical principles under which the journal operates.

### Animals

Wild-type C57Bl/6J (Envigo, UK) female (10–16 weeks old) and male (12–26 weeks old) mice were mated and discovery of a copulation plug was used to define embryonic day (E) 0.5 (term = E19.5). Mice were provided with nesting material and communally housed (with the exception of stud males that were individually housed) in individually ventilated cages under a constant 12 h light/dark cycle at 21–23°C with free access to food (BK001 diet, Special Dietary Services, Essex, UK) and water (Hydropac, Denver, US). Pregnant female mice were euthanized by cervical dislocation alone, or terminal anesthesia followed by exsanguination (cardiac puncture) and cervical dislocation (only for unidirectional maternofetal calcium clearances measures; see below) appropriate under ASPA schedule 1. Following euthanasia a laparotomy and hysterotomy was performed. All fetuses were rapidly killed by cervical dislocation.

**Figure 1:**
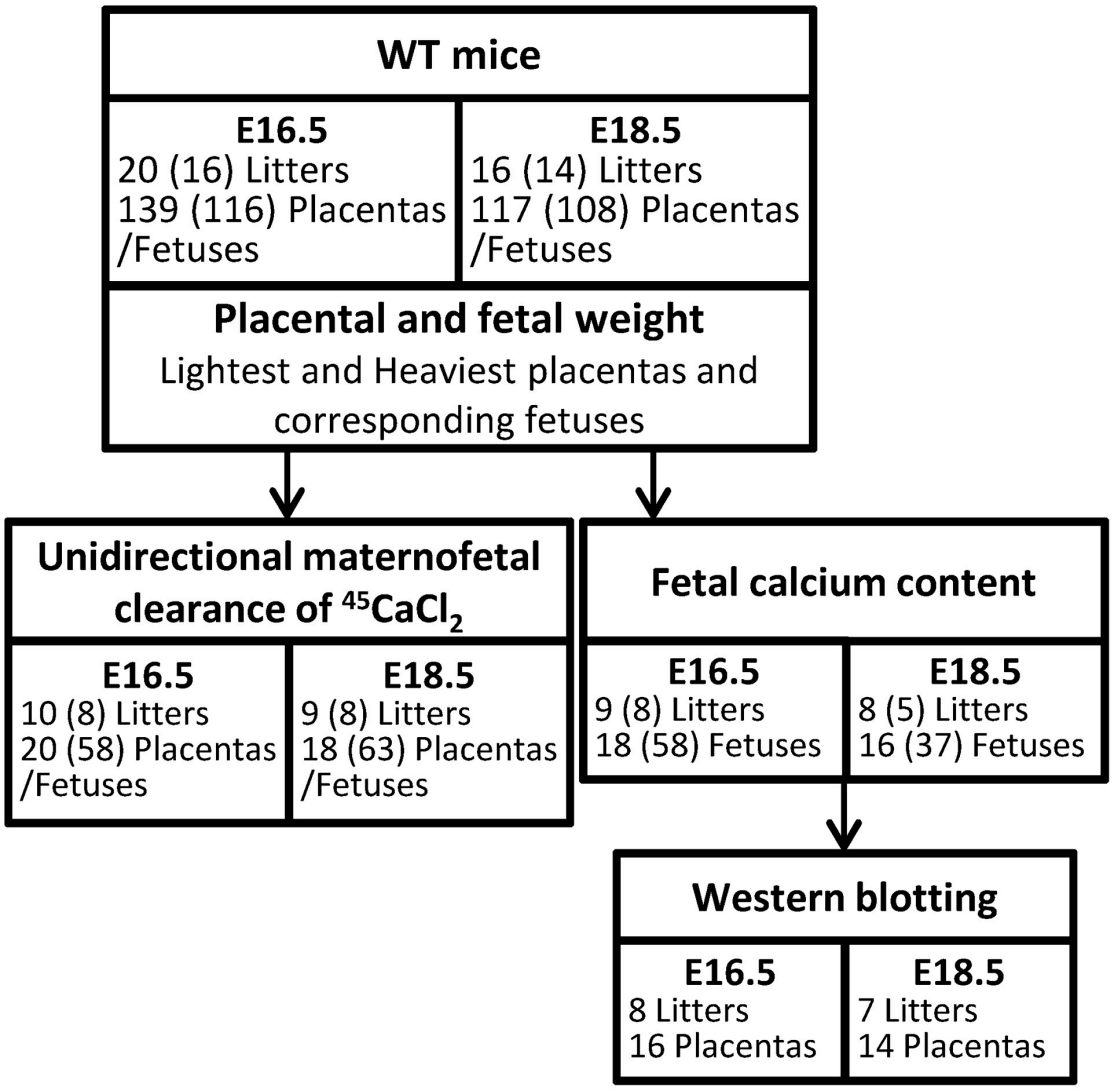
Schematic diagram of methods used in wild-type (WT) mice at embryonic day (E) 16.5 and E18.5. Numbers stated represent those litters used for lightest vs. heaviest analyses. (N) represents the number of litters used to analyse litter averages of female vs. male fetuses. The lower number represents only those litters with at least 2 females and 2 males.

On E16.5 (*N* = 20 litters; *n* = 139 placentas and fetuses) and E18.5 (*N* = 16 litters; *n* = 117 placentas and fetuses), pregnant WT females were euthanized and fetuses and placentas were rapidly harvested, blotted and wet weights measured. Median litter sizes were 7 [range 4–9] and 8 [4–10] at E16.5 and E18.5, respectively. Following the identification of the lightest and heaviest placentas in a litter, all placentas and fetuses (excluding those used for the unidirectional maternofetal calcium clearance experiments; see below) were snap frozen and stored at −80°C (Figure 1). Fetal tail tips were collected from all fetuses and stored at −20°C for sex determination. The aim of the study, comparing lightest vs. heaviest placentas and fetal sex within a single litter, meant that randomization or blinding of the samples was not possible. Comparisons of placental weight, fetal weight and fetal weight: placental weight ratio were made between the lightest and heaviest placentas from each litter at E16.5 (*n* = 20 lightest vs. *n* = 20 heaviest) and at E18.5 (*n* = 16 lightest vs. *n* = 16 heaviest), and between litter averages for females and males at E16.5 (*N* = 16 litters; *n* = 58 and *n* = 58, respectively) and at E18.5 (*N* = 14 litters; *n* = 59 and *n* = 49). There are fewer litters in the male vs. female comparisons as litter averages were calculated only for those litters with at least 2 males and 2 females.

### Determining fetal sex

Fetal sex was determined for all fetuses according to a previously published genotyping protocol (Kunieda et al., 1992); genomic DNA was extracted from fetal tail tips using DirectPCR lysis reagent (mouse tail) (Bioquote, York, UK) containing proteinase K (Qiagen, Manchester, UK) and MyTaq Red Mix (Bioline Ltd, London, UK) with primers specific to gene sequences for *SRY* (Y chromosome; 404 bp; *SRY2* 5′-TCTTAAACTCTGAAGAAGAGAC-3′, *SRY4* 5′-GTCTTGCCTGTATGTGATCG-3′) and *NDS* (X chromosome; 244 bp; *NDS3* 5′-GAGTGCCTCATCTATACTTACAG-3′, *NDS4* 5′-TCTAGTTCATTGTTGATTAGTTGC-3′) (Kunieda et al., 1992). PCR conditions were as follows; 5 min denaturation at 94°C, 35 cycles of 1 min at 94°C, 1 min at 55°C, 1 min at 72°C and 10 min final extension at 72°C. Samples were run on a 2% agarose gel and bands visualized using an InGenius transilluminator (Sygene Bio, Cambridge, UK).

### Unidirectional maternofetal clearance of ^45^Ca across the intact placenta (^Ca^K_mf_)

Unidirectional maternofetal ^45^Ca clearance (^Ca^K_mf_) was assessed across the intact placenta at E16.5 (*N* = 10 litters) and E18.5 (*N* = 9 litters) as previously described (Flexner and Pohl, 1941; Bond et al., 2008; Dilworth et al., 2010). The procedure was carried out according to the local rules following the Ionising Radiations Regulations (1999) approved by the Manchester University Foundation NHS Trust Radiation Protection Advisor. All users were professionally trained and certified to work with radiation and had read, signed and adhered to the ionizing radiation risk assessments and standard operating procedures in place. Mice were anesthetized (1 part Hypnorm, VetaPharma Ltd, Leeds, UK: 2 parts water: 1 part Midazolam, Roche, UK) by i.p. injection. Following injection of 2 μCi ^45^CaCl_2_ (Perkin Elmer, UK) into the tail vein of the anesthetized dam, a cardiac puncture was performed 2–3 min post-infusion which exsanguinated the dam. All fetuses and placentas were collected, assessed for total radiolabel accumulation and compared to a maternal plasma ^45^Ca disappearance curve (see below). ^Ca^K_mf_ was calculated as previously described (Dilworth et al., 2010). All clearance experiments were carried out between the hours of 08:00 and 12:00 in the same theater room. Comparisons were made between: the lightest and heaviest placentas from each litter at E16.5 (*n* = 10 lightest vs. *n* = 10 heaviest) and at E18.5 (*n* = 9 lightest vs. *n* = 9 heaviest); and between litter averages for female and males at E16.5 (*N* = 8 litters; *n* = 28 and *n* = 30, respectively) and at E18.5 (*N* = 8 litters; *n* = 34 and *n* = 29). Only those litters with at least 2 males and 2 females were included in the female vs. male comparisons.

### Maternal plasma ^45^Ca disappearance curve following injection into the maternal circulation

Since repeated blood sampling, from time of isotope injection to time of sacrifice, is not possible in the mouse due to a low circulating blood volume, a maternal plasma disappearance curve was constructed from dams at either E16.5 (*N* = 13 litters) or E18.5 (*N* = 13 litters) and fitted to a one-phase exponential decay model (*r*^2^ > 0.5), as described previously (Bond et al., 2006).

### Fetal calcium content

As previously described (Bond et al., 2008), fetal calcium content was measured in all fetuses at E16.5 (*N* = 9 litters) and E18.5 (*N* = 8 litters) by inductively coupled plasma atomic emission spectroscopy (Perkin-Elmer Optima 5300 dual view ICP-AES) following ashing of fetuses overnight at 800°C in a Gallenkamp Furnace (Gallenkamp & Co, UK). Comparisons were made between: the lightest and heaviest placentas from each litter at E16.5 (*n* = 9 lightest vs. *n* = 9 heaviest) and at E18.5 (*n* = 8 lightest vs. *n* = 8 heaviest); and between litter averages for female and males at E16.5 (*N* = 8 litters; *n* = 30 and *n* = 28, respectively) and at E18.5 (*N* = 8 litters; *n* = 19 and *n* = 18). Only litters with at least 2 males and 2 females were included in the female vs. male comparisons.

### Protein expression

The lightest and heaviest placentas harvested at E16.5 (*N* = 8 litters) and E18.5 (*N* = 7 litters) were homogenized and processed as described previously (Dilworth et al., 2010); briefly, whole homogenates were separated, by means of centrifugation, into a membrane fraction (containing the MVM, BM, and organelle plasma membranes) and a cytosolic fraction. SDS-PAGE was performed followed by electrotransfer to Immobilon-FL PVDF membranes (Millipore UK Ltd, Watford, UK). Primary antibodies included: rabbit polyclonal antibodies for TRPV6 (1 μg/ml; H-90, sc-28763; Santa Cruz Biotechnology, c/o Insight Biotechnology Ltd, Wembley, UK), calbindin-D_9K_ (1:1000; CB9; SWANT, Marly, Switzerland) and calcium ATPase isoform 1 (PMCA1, 1.3 μg/ml; 69; SWANT). β-actin (0.5 μg/ml; ab8227; Abcam, Cambridge, UK) was used as a loading control; no difference was observed in β-actin expression between groups. Negative controls were by omission of primary antibody. Immunoreactive species were detected with fluorescent-conjugated secondary antibodies (Li-COR Biosciences, Cambridge, UK) and membranes imaged using an Odyssey Sa Infrared Imaging System (Li-COR). Signal density was measured using Image Studio Lite (Li-COR). All signals were in the linear range of detection. Membrane fractions were used for assessing the expression of TRPV6 and PMCA1 and the cytosolic fraction was used for assessing calbindin-D_9K_ expression. Comparisons were made between the lightest and heaviest placentas at E16.5 and at E18.5 separately.

### Statistical analysis

Data are presented as the lightest placenta as a percentage of the heaviest in a litter (dotted line is heaviest = 100%), and also as the litter average of females as a percentage of the litter average of males (dotted line is male = 100%), or median [range] where the experimental *N* = number of litters, *n* = number of placentas or fetuses. When considering effects of fetal sex, litter averages were used thus inclusion criteria meant that litters had to have a minimum of 2 females and 2 males.

The solid line on graphs represents the median value. To calculate sample sizes per experiment, we utilized data from previous studies (Dilworth et al., 2010) and employed an Altman's nomogram to calculate the standardized difference based upon 90% statistical power at a 5% significance level. Data were analyzed by Wilcoxon matched-pairs signed-rank test or Mann Whitney test. *P* < 0.05 was considered statistically significant.

## Results

### Placental and fetal weight in WT litters

At E16.5, 75% (15/20) of the placentas in the lightest groups were from females; this figure increased to 100% at E18.5. At E16.5, 75% (15/20) of the placentas in the heaviest group were from males; this figure was 81% (13/16) at E18.5. At E16.5 (*N* = 20) and E18.5 (*N* = 16), the weights of the lightest placentas were 78 and 79% of the heaviest placentas (Table 1). Fetuses of the lightest placentas weighed less than those from the heaviest placentas at E16.5 and E18.5. Fetal weight histograms were constructed and a non-linear regression performed (Gaussian distribution) from which individualized fetal weight centiles were calculated. Fetal weight centiles were lower in the lightest compared to heaviest placenta group at E16.5 (median 27.3 vs. 69.8; *P* < 0.01) and E18.5 (31.1 vs. 74.8; *P* < 0.05) but were still within the normal range of fetal weight (10–90th fetal weight centile). Fetal weight: placental weight (F:P) ratio was used as a proxy of placental efficiency (gram of fetus produced per gram of placenta) and was higher in the lightest vs. heaviest placenta group at E16.5 and E18.5.

**Table 1:** Placental weight, fetal weight and fetal weight: placental weight (F:P) ratio in wild-type (WT) mice at embryonic day (E) 16.5 and E18.5.

	**E16.5**				**E18.5**			
	**Lightest**	**Heaviest**	**Lightest/Heaviest (%)**	***P*-value**	**Lightest**	**Heaviest**	**Lightest/Heaviest (%)**	***P*-value**
Placental weight (g)	0.078 (0.066–0.116)	0.097 (0.092–0.152)	78.0 (68.0–87.0)	< 0.0001	0.071 (0.061–0.080)	0.092 (0.083–0.097)	79.0 (72.0–89.0)	< 0.0001
Fetal weight (g)	0.565 (0.446–0.782)	0.643 (0.491–0.760)	88.5 (74.0–122.0)	0.0007	1.164 (1.075–1.262)	1.249 (1.155–1.331)	93.5 (83.0–108.0)	0.01
F:P ratio	7.2 (4.9–10.2)	6.6 (3.9–7.8)	113.5 (95.0–152.0)	0.0002	16.2 (14.6–19.0)	13.9 (12.6–15.1)	118.0 (108.0–133.0)	< 0.0001
	**Female**	**Male**	**Female/Male (%)**	***P***-**value**	**Female**	**Male**	**Female/Male (%)**	***P***-**value**
Placental weight (g)	0.085 (0.077–0.104)	0.092 (0.085–0.109)	93.1 (82.7–100.9)	< 0.0001	0.076 (0.068–0.083)	0.084 (0.077–0.089)	92.0 (84.1–98.1)	0.0001
Fetal weight (g)	0.592 (0.480–0.671)	0.628 (0.497–0.668)	95.0 (86.4–100.8)	0.0003	1.193 (1.105–1.238)	1.225 (1.021–1.275)	97.5 (93.0–108.3)	0.09
F:P ratio	7.0 (5.1–7.9)	6.9 (5.3–7.6)	101.9 (93.7–112.2)	0.20	15.7 (14.1–17.1)	14.5 (12.7–15.7)	107.5 (101.8–112.1)	0.0001

*Data are median (range) or presented as the lightest placenta as a percentage of the heaviest in a litter (lightest/heaviest (%) column) or as the litter average of females as a percentage of the litter average of males (female/male (%) column). Data are analyzed by Wilcoxon matched-pairs signed rank test (lightest vs. heaviest; female vs. male). P < 0.05 was considered statistically significant*.

At E16.5 (*N* = 16) and E18.5 (*N* = 14), placental weights from female fetuses (litter average of litters containing at least 2 female and 2 male fetuses) were 93 and 92% of those from male fetuses (Table 1). Weights of female fetuses were lower than their male littermates at E16.5 but were similar at E18.5. Fetal weight centiles, though still in the normal range, were lower for female fetuses than for male fetuses at E16.5 (37.5 vs. 58.7; *P* < 0.0001) and at E18.5 (46.0 vs. 63.5; *P* < 0.05). F:P weight ratio was similar in males and females at E16.5 but higher in females at E18.5 (*P* < 0.01).

### Fetal calcium content and unidirectional maternofetal calcium clearance

**Figure 2:**
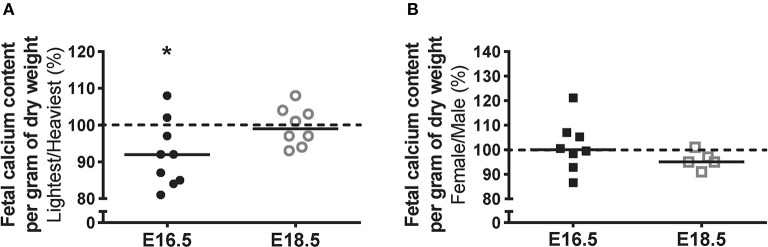
Fetal calcium content (an estimate of net placental calcium flux) at embryonic day (E)16.5 and E18.5 expressed as the lightest as a percentage of the heaviest placentas **(A)** and as the litter average female as a percentage of male **(B)** within the same litter. For **(A)**, raw values (mmol/g fetal dry weight) were as follows (median [range]): E16.5 (*N* = 9) lightest (*n* = 9; 0.19 [0.16–0.21]) and heaviest (*n* = 9; 0.21 [0.16–0.23]); and E18.5 (*N* = 8) lightest (*n* = 8; 0.37 [0.34–0.38]) and heaviest (*n* = 8; 0.37 [0.32–0.40]). For **(B)**, raw values were: E16.5 (*N* = 8) female (*n* = 30; 0.20 [0.17–0.23]) and male (*n* = 28; 0.20 [0.17–0.23]); and E18.5 (*N* = 5) female (*n* = 19; 0.37 [0.35–0.37]) and male (*n* = 18; 0.38 [0.37–0.38]). Black line = median; dotted line 100% = heaviest placenta **(A)** or male **(B)**. ^*^P < 0.05; Wilcoxon matched-pairs signed-rank test.

Fetal calcium content (Figure 2A) was used in the current study as an estimate of net placental calcium flux. Calcium content, expressed as mmol/g fetal dry weight, was 8% lower in fetuses from the lightest placentas compared to those from the heaviest placentas at E16.5 (*P* < 0.05). At E18.5, fetal calcium content was comparable between the lightest compared to the heaviest placenta group. There was no significant difference in fetal calcium content at either gestational age between female and male littermates (Figure 2B).

**Figure 3:**
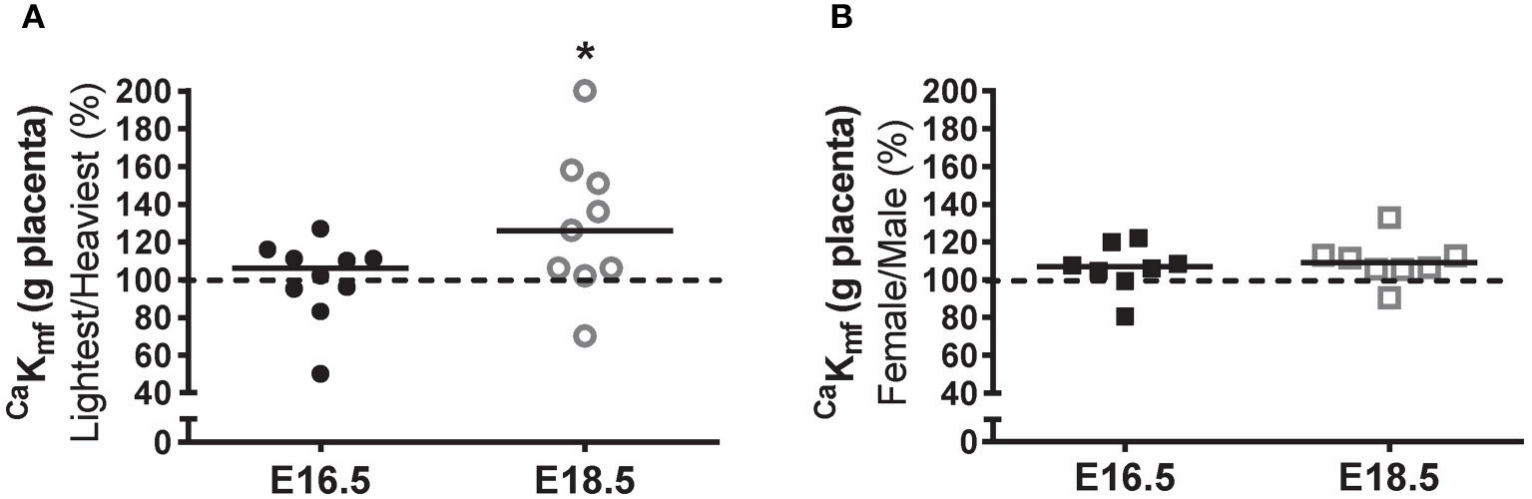
Unidirectional maternofetal calcium clearance at embryonic days (E)16.5 and E18.5 expressed as the lightest as a percentage of the heaviest placentas **(A)** and as litter average female as a percentage of male **(B)** within the same litter. For **(A)**, raw values (^Ca^K_mf;_ μL/min/g placenta) were as follows (median [range]): E16.5 (*N* = 10) lightest (*n* = 10; 89.1 [47.6–129.3]) and heaviest (*n* = 10; 99.1 [43.3–110.3]); and E18.5 (*N* = 9) lightest (*n* = 9; 104.1 [49.9–345.0]) and heaviest (*n* = 9; 71.2 [60.0–339.4]). For **(B)**, raw values (^Ca^K_mf;_ μL/min/g placenta) were as follows: E16.5 (*N* = 8) female (*n* = 28; 98.3 [45.7–120.1]) and male (*n* = 30; 89.6 [56.7–105.8]); E18.5 (*N* = 8) female (*n* = 34; 107.4 [56.5–335.6]) and male (*n* = 29; 99.0 [62.5–315.3]). Black line = median; dotted line 100% = heaviest placenta **(A)** or male **(B)**. ^*^P < 0.05; Wilcoxon matched-pairs signed-rank test.

Unidirectional maternofetal clearance of calcium per gram of placenta (μL/min/g placenta) was not different between the lightest and heaviest placenta groups at E16.5 but was significantly higher in the lightest compared to the heaviest placentas at E18.5 (*P* < 0.05; Figure 3A). Unidirectional maternofetal calcium clearance was not different between female and male fetuses at E16.5 and had a tendency to be higher at E18.5 (*P* = 0.055; Figure 3B) in placentas from female vs. male fetuses.

### Expression of placental calcium transport pathway components

**Figure 4:**
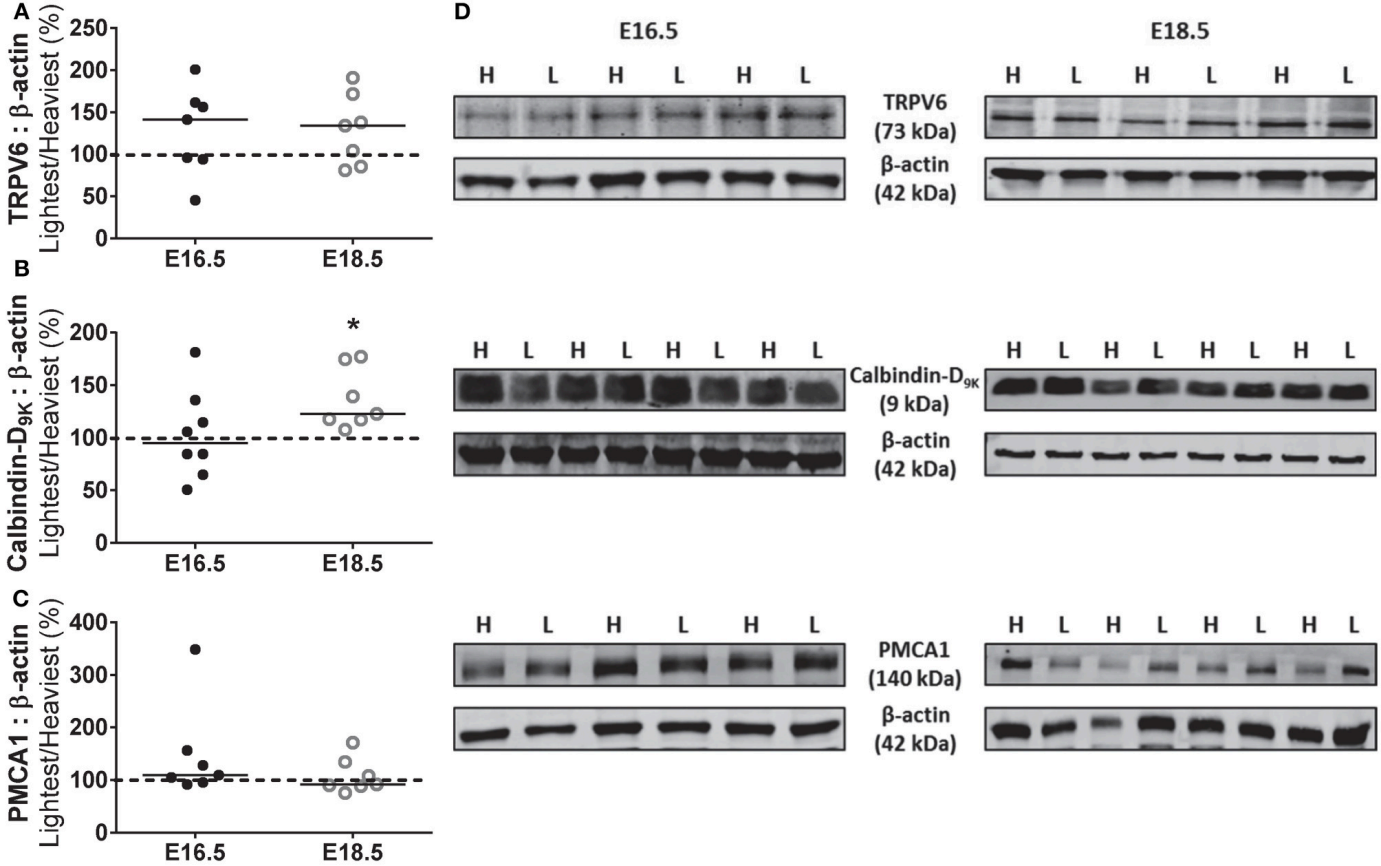
Expression of placental calcium transport pathway components. Placental protein expression (expressed as percentage of lightest (L) vs. heaviest (H) placenta within the same litter) of transient receptor potential vanniloid type 6, TRPV6 **(A)**, calbindin-D_9K_ **(B)** and plasma membrane calcium ATPase, PMCA1 **(C)** at either embryonic day (E) 16.5 (*N* = 7–8) or E18.5 (*N* = 7–8). Densitometric analysis is expressed as a ratio to β-actin signal. **(D)** Representative Western blots of TRPV6, calbindin-D_9K_ and PMCA1 at E16.5 and E18.5 with the corresponding loading control. Black line = median; dotted line 100% = heaviest placenta. ^*^P < 0.05; Wilcoxon matched-pairs signed-rank test.

Western blotting was used to determine whether the increased unidirectional maternofetal calcium clearance observed in the lightest placentas was associated with changes in placental expression of components of the calcium transport pathway (as described earlier). In the protein expression studies, the lightest placentas were all from female fetuses whilst the heaviest placentas were predominantly from male fetuses (5/8 [63%] at E16.5 and 5/7 [71%] at E18.5). At E16.5, there was no difference in the expression of calcium transporters, TRPV6 and PMCA1, and the calcium binding protein, calbindin-D_9K_, between the lightest and heaviest placentas of a WT litter. There was also no difference in expression of TRPV6 and PMCA1 between the lightest and heaviest placentas of a WT litter at E18.5. However, expression of calbindin-D_9k_ was increased by 22% in the lightest compared to the heaviest placenta of the same litter at E18.5 (*P* < 0.05; Figure 4, Supplementary Figures 1–3).

## Discussion

In support of our hypothesis, the lightest placenta in a normal (WT) mouse litter had a higher maternofetal clearance of calcium (per gram placenta) than the heaviest placenta near term. We suggest this increased transfer efficiency, coinciding with increased placental calbindin-D_9K_ expression, is an example of a placental adaptation which promotes fetal calcium acquisition despite a relatively small placental size. The normalization of fetal calcium accretion, relative to fetal size, by E18.5, following a reduction at E16.5, may be indicative of a fetus signaling to its placenta, by as yet unknown mechanisms, to increase maternofetal transfer of calcium. This adaptation by a small placenta is also likely to prevent fetal bone mineralisation being compromised; this is of particular importance as poor fetal provision of calcium *in utero* has been linked with an increased risk of developing osteoporosis later in life (Tobias and Cooper, 2004). These data suggest that in normal pregnancy, calcium transport by a smaller placenta is adaptively up-regulated to ensure appropriate calcium supply to the fetus and adequate fetal growth.

As the lightest placentas were predominantly from female fetuses (75% at E16.5 and 100% at E18.5), it is possible that the effects seen were due not just to differences in placental size, but also due to sex-dependent effects. To investigate this, we compared female and male fetuses within each litter to determine whether fetal sex had an impact on the adaptive changes observed in placental calcium transfer. On average, females had lower fetal and placental weights than their male littermates. Females also demonstrated a higher F:P ratio vs. males, indicating greater placental efficiency in female fetuses; this may account for the comparable birthweight to males near term. Fetal calcium content and placental calcium transfer, however, were comparable in females and males at both gestational ages, although there was a trend toward increased ^Ca^K_mf_ in females vs. males at E18.5 (*P* = 0.055). This indicates that the lightest placentas' adaptive increase in unidirectional maternofetal calcium transfer, in apparent response to reduced fetal calcium content, is unlikely to be solely explained by the disparity between males and females that make up the lightest and heaviest groups.

In WT mouse litters, the lightest placentas were, on average, more than 20% lighter than the heaviest at both gestational ages, whilst the difference in the weight of fetuses with the lightest compared to heaviest placentas fell with increasing gestational age; a reduction of 11% at E16.5 and 6% at E18.5. This is indicative of a fetus catching up to its sibling close to term, likely due to increased rates of placental nutrient transfer, as described in this and another study (Coan et al., 2008). In the study by Coan et al. (2008), differences in fetal weights in the lightest vs. heaviest placenta groups were similar to those reported here (12% reduction at E15.5 and 4% [not statistically significant] at E18.5), supporting the concept of placental adaptation. Fetuses from the lightest and heaviest placentas were within what would be considered a “normal” fetal weight range, i.e., between the 10 and 90th centiles, suggesting a placenta that adapts to prevent both fetal undergrowth in the case of a small placenta and prevents fetal overgrowth in the case of a relatively large placenta.

In the study by Coan et al. (2008), morphological differences were described in lightest vs. heaviest placentas in WT (C57Bl/6J) mice; these included a reduction in the junctional zone and thus a relative increase in the labyrinthine zone (important for nutrient transfer) which led to an increased surface area for exchange. At E18.5, these differences were no longer apparent. This led to the conclusion that placental adaptations were morphological in nature earlier in gestation but that functional adaptations were more important near term, as evidenced by increased rates of System A transfer in lightest placentas and increased placental expression of the gene encoding the sodium-dependent neutral amino acid transporter-2 transporter (Coan et al., 2008). These adaptations resulted in an increased F:P ratio (Coan et al., 2008) and indeed we also observed an increased F:P ratio in the lightest vs. heaviest placentas at E16.5 (16%) and E18.5 (19%). Fetal sex was not reported by Coan and colleagues and the relative contributions of placental size and fetal sex on adaptive increases in placental System A transport have yet to be established.

In rodents, increased placental calcium transfer and fetal accrual toward term (E15–19) is accompanied by a 14 and 5 fold increase in TRPV6 and calbindin-D_9K_ mRNA expression, respectively (Mathieu et al., 1989; Glazier et al., 1992; An et al., 2004; Suzuki et al., 2008). In the current study, increased maternofetal clearance of calcium in the lightest placentas was not associated with higher expression of TRPV6 or PMCA1. However, calbindin-D_9K_ protein expression was increased in the lightest vs. heaviest WT placentas at E18.5 suggesting more capacity for calcium binding and increased shuttling to the BM for transfer to the fetus via PMCA. Therefore, calbindin-D_9K_ is potentially important in adaptive mechanisms. It should be noted that one limitation of this analysis is that we did not separate the junctional and labyrinthine zones prior to extracting membrane and cytosolic fractions. It is known that the mouse placenta has functionally distinct regions and the labyrinthine zone, important for nutrient transfer, increases in size relative to the size of the junctional zone near term (Coan et al., 2008). This alteration in the relative proportions of the zones enables increased nutrient transfer capacity near term. Future work involving calciotropic proteins should focus primarily on the labyrinthine zone and the distribution of other calcium binding proteins, such as calbindin-D_28K_ and calmodulin, within the cytosolic fraction. Protein expression analyses between lightest vs. heaviest placentas were predominantly assessments of placentas from female vs. male fetuses (5/8 litters at E16.5 and 5/7 litters at E18.5). In those litters that were not comparisons of male and female but were female to female comparisons (3/8 litters at E16.5 and 2/7 litters at E18.5), calbindin-D_9K_ expression in the lightest as a percentage of the heaviest placenta was 51%, 106% and 115% at E16.5, and, 108% and 117% at E18.5. These values, at E18.5 especially, appear broadly in line with the male vs. female comparisons but we cannot discount the possibility that the increased expression of calbindin-D_9K_ in the lightest placentas at E18.5 may be due, in part, to fetal sex.

In a well characterized mouse model of late onset FGR, the placental-specific insulin-like growth factor 2 knock out (P0) mouse (Constância et al., 2005), there is a reduction in placental weight from E14 which precedes the reduction in fetal weight (E19). This delay in FGR is thought to be due to an earlier adaptive response by the placenta, whereby placental System A amino acid transport is increased, thus helping to maintain fetal growth on a normal trajectory. By E19 (equivalent to E18.5 in the current study), this adaptation is no longer apparent which, coupled with a decreased placental permeability, results in late onset FGR in the P0 mouse (Constância et al., 2002; Sibley et al., 2004; Dilworth et al., 2011). This reduction in System A transport and reduced permeability are akin to findings in human FGR (Glazier et al., 1997; Jansson et al., 1998; Mayhew et al., 2003; Shibata et al., 2008). By contrast, adaptive up-regulation of calcium transport in these mice was observed at the end of gestation (Dilworth et al., 2010). This may, at least in part, reflect the fact that fetal skeletal mineralisation occurs late in gestation in rodents (Comar, 1956; Glazier et al., 1992). Increased placental calcium transport in the P0 mouse was associated with altered calbindin-D_9K_ protein expression [lower in P0 at E17 (equivalent to E16.5 in the current study), but comparable at E19], indicating calbindin-D_9K_ contributes to an adaptive response in FGR, in common with the current study. Like P0 mice (Dilworth et al., 2010), WT fetuses from the lightest placenta in a litter, have reduced fetal calcium content, relative to fetal size, at E16.5 which is normalized by E18.5, presumably due to the increased maternofetal transfer of calcium by these lightest placentas. This timeline suggests that there may be as yet unidentified fetal signals which are able to elicit changes in placental function, as evidenced here by the increased placental ^Ca^K_mf_, that ultimately leads to increased fetal skeletal mineralisation and growth of the fetus.

Calbindin-D_9K_ is implicated as a mediator of placental adaptation in calcium transfer both in this and our previous studies in the P0 mouse (Dilworth et al., 2010). However, evidence from the calbindin-D_9K_ knockout mouse indicates that other candidates might also be involved (Lee et al., 2007; Koo et al., 2012). In the absence of calbindin-D_9K_, placental transfer of calcium was normal as other proteins, in particular calbindin-D_28K_, TRPV5/6 and the sodium-calcium exchanger appeared to compensate, all showing increased expression (Koo et al., 2012). Calbindin-D_9K_, -D_28K_ and TRPV6 have all been identified in placentas of women (Belkacemi et al., 2003, 2004; Haché et al., 2011; Yang et al., 2013) but there is limited data on calcium transport in human placenta. In the study by Haché et al. (2011), calcium transport, as assessed using a primary trophoblast cell culture model in which cells were isolated from term placentas, was reported to be lower in pre-eclampsia vs. normal pregnancy. Yang et al. (2013) suggested that expression of TRPV6 and PMCA1 was increased in placentas from pre-eclamptic women (Yang et al., 2013). Whilst there are a lack of comparative studies in FGR, Strid and colleagues did demonstrate, in women, that ATP-dependent transfer of calcium, via PMCA, is increased in the basal membrane of FGR vs. normal pregnancies (Strid et al., 2003). This may be an example of a placental adaptation, thus maintaining appropriate fetal calcium provision despite a small placenta, as we observed both in the P0 knockout mice (Dilworth et al., 2010) and in the current study, where a small, but normal, placenta was observed. The increased ^Ca^K_mf_ described in the current study is comparable to our studies in another mouse model of FGR, the parathyroid hormone related protein (PTHrP) knockout mouse. PTHrP knockout mice demonstrated increased maternofetal calcium flux as measured *in vivo* and using a dual perfusion model (Bond et al., 2006, 2008), though this contrasts with previous reports suggesting a reduced maternofetal calcium flux vs. WT (Kovacs et al., 1996). PTHrP is essential for normal functional development of the mouse placenta (Duval et al., 2017) and has been shown to stimulate PMCA in BM preparations from human placenta (Strid et al., 2002). Studies that assess expression of PTHrP in the placenta and fetus between lightest and heaviest placentas may be worthwhile in the future, as are studies which assess maternofetal calcium transfer in human FGR. Whilst ethical considerations prevent these types of studies *in vivo*, novel culture systems which mimic the placental barrier *in vitro* may prove useful in this regard (Blundell et al., 2016).

Despite the predominance of female fetuses in the lightest placenta group, fetal calcium content did not alter according to fetal sex. However, there was a trend toward increased unidirectional maternofetal calcium transfer in females vs. males near term, consistent with the increased F:P ratio at E18.5 in females vs. males. This increased F:P ratio suggests that functional adaptations in the placenta prevent female fetuses being smaller than their male littermates near term and that, whilst not quite significant in terms of calcium, other nutrient transport systems, such as system A, are worthy of investigation. The current study and recent collated data (Clifton, 2010) demonstrate the importance of examining the effects of fetal sex when considering placental function and development. To further investigate the placental adaptive changes observed in the current study, comparisons would be required between lightest and heaviest placentas of females and lightest and heaviest placentas of males in the same litter. However, in this inbred C57Bl/6J (WT) mouse litter, relatively small litter sizes compared to outbred strains, and a limited disparity in placental weights, make this problematic. Examining litters of an outbred mouse strain or in rats might provide the opportunity in which to directly test the relative contribution of fetal sex in comparison to placental size.

This study has shown that calcium transfer across lightest placentas in WT litters is adaptively up-regulated so that all fetuses, whether with lighter or heavier placentas, demonstrate an appropriate level of calcium accretion relative to their size. This will likely have important implications in terms of appropriate fetal skeletal mineralisation. The timeline of this adaptation is similar to those changes in placental nutrient transfer observed in P0 knockout mice (Dilworth et al., 2010) and points to a role for fetal nutrient demand in driving this adaptation via calcium binding proteins. Fetal sex does not appear to be a major contributor to adaptations in maternofetal calcium transfer. These data show that placental adaptations are an important feature of normal fetal growth and that further work is required to fully elucidate the underlying regulatory mechanisms. Better understanding of these mechanisms, and of those that occur in FGR, may provide novel therapeutic avenues to exploit in cases of poor fetal growth.

## Author contributions

Conception or design of the work: CH, CS, SG, and MD. Acquisition, analysis or interpretation of data for the work: CH, LR, CS, SG, and MD. Drafting the work or revising it critically for important intellectual content: CH, LR, CS, SG, and MD. All authors approved the final version of the manuscript and agree to be accountable for all aspects of the work ensuring that questions related to the accuracy or integrity of any part of the work are appropriately investigated and resolved. All persons designated as authors qualify for authorship, and all those who qualify for authorship are listed.

### Conflict of interest statement

The authors declare that the research was conducted in the absence of any commercial or financial relationships that could be construed as a potential conflict of interest.
