# Supplementary figures and images for: Adaptations in Maternofetal Calcium Transport in Relation to Placental Size and Fetal Sex in Mice
Source: Front Physiol. 2017 Dec 12;8:1050. doi: 10.3389/fphys.2017.01050 (PMC5732954; doi:10.3389/fphys.2017.01050)

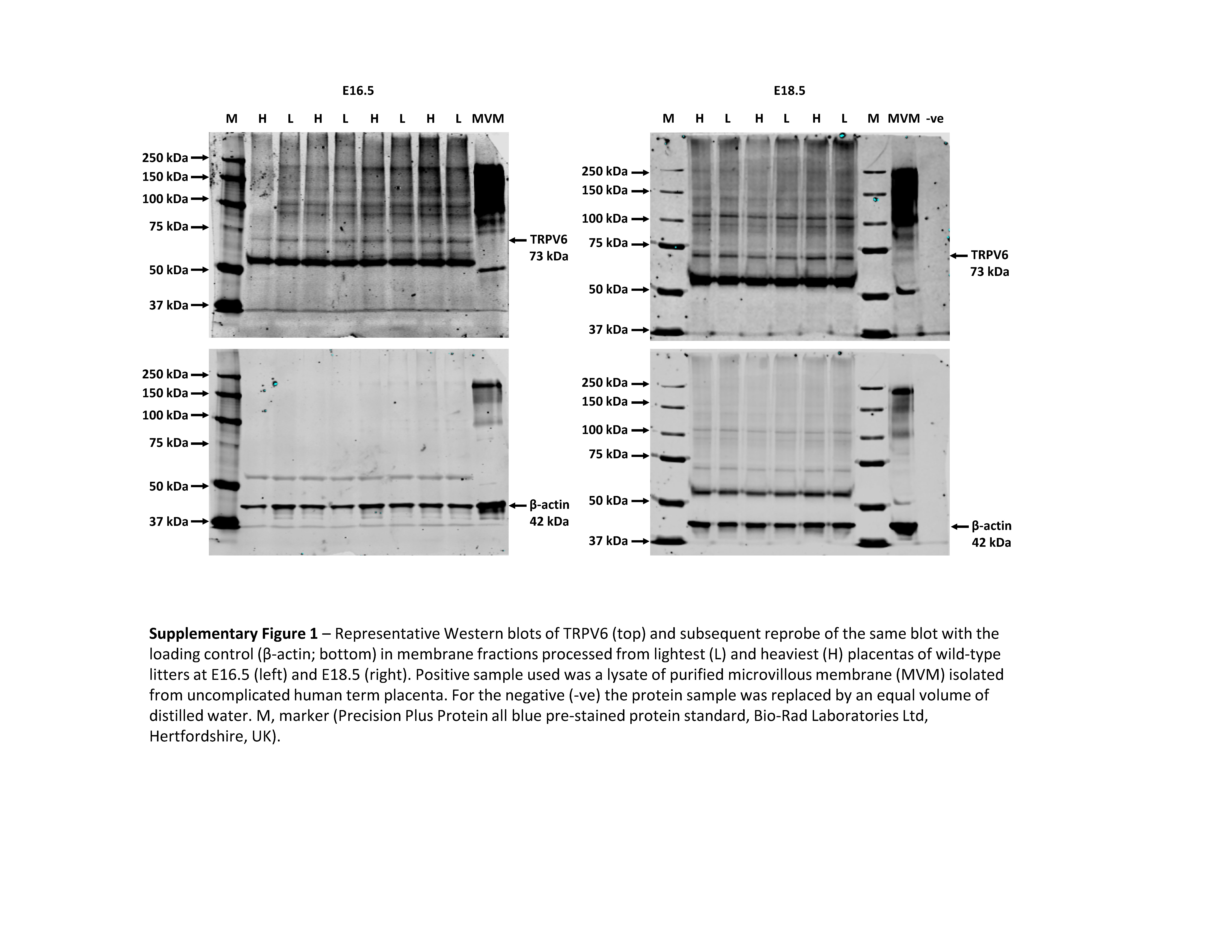

Supplement: Supplementary file 1 [file Image1.jpg]

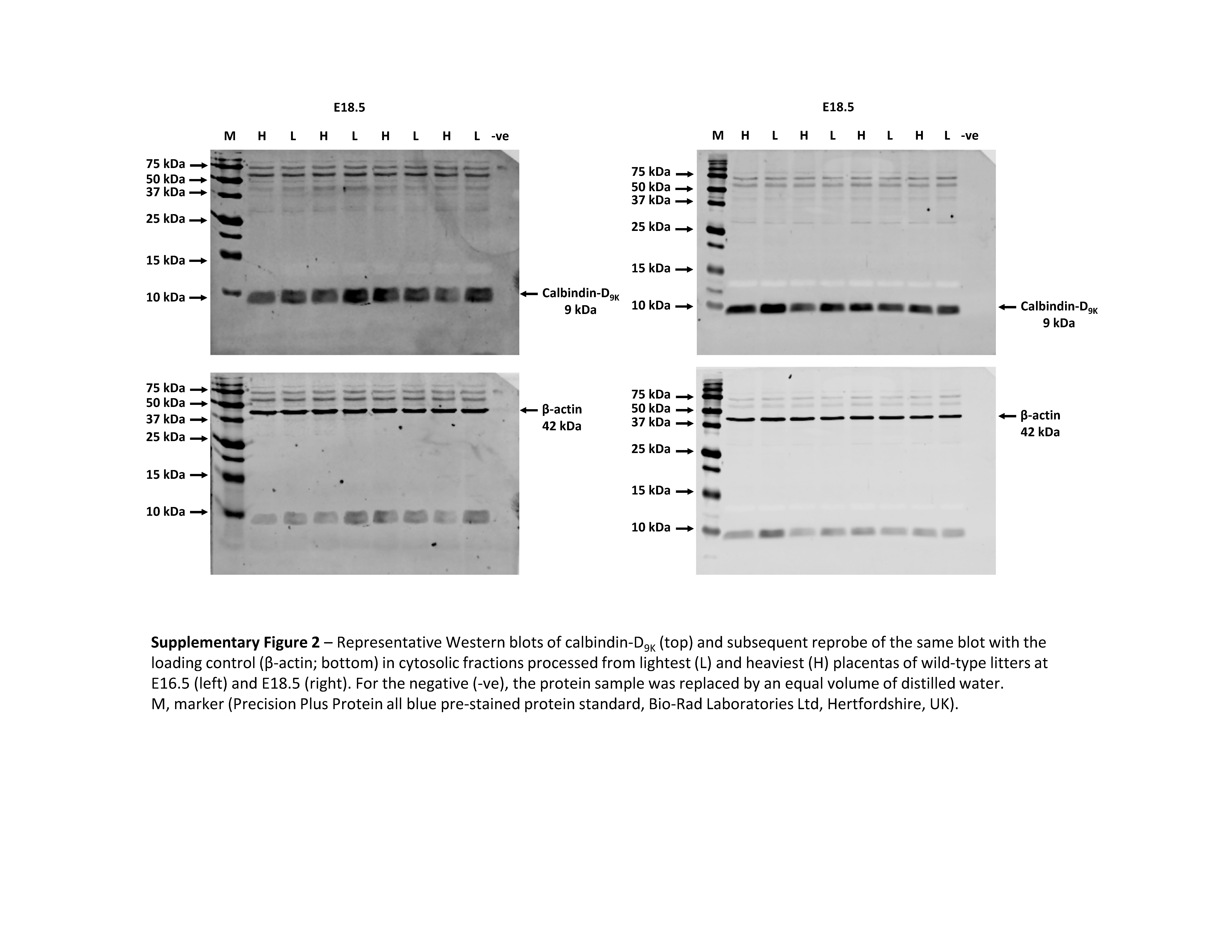

Supplement: Supplementary file 2 [file Image2.jpg]

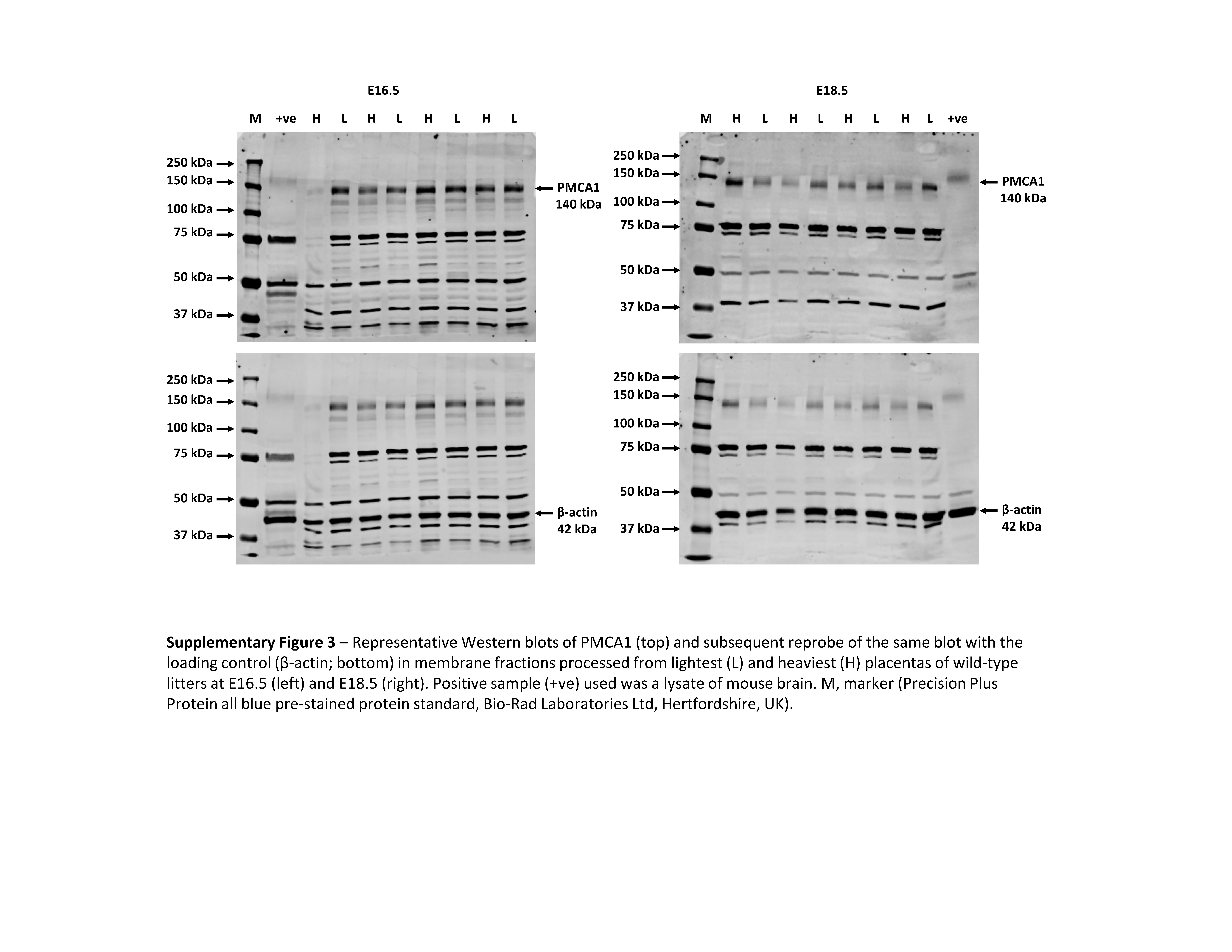

Supplement: Supplementary file 3 [file Image3.jpg]
